# Review of pharmacological therapies in fibromyalgia syndrome

**DOI:** 10.1186/ar4441

**Published:** 2014-01-17

**Authors:** Winfried Häuser, Brian Walitt, Mary-Ann Fitzcharles, Claudia Sommer

**Affiliations:** 1 Department of Internal Medicine I, Klinikum Saarbrücken, Winterberg 1, D-66119, Saarbrücken, Germany; 2 Department Psychosomatic Medicine and Psychotherapy, Technische Universität München, Langerstr. 3, D-81675, München, Germany; 3 Georgetown University Medical Center, Washington Hospital Center, 110 Irving Street, Washington, DC 20010, USA; 4 Division of Rheumatology, McGill University Health Centre; Alan Edwards Pain Management Unit, McGill University Health Centre, Montreal H3G 1A4, Canada; 5 Department of Neurology, University of Würzburg, Josef-Schneider-Str. 11, D-97080, Würzburg, Germany

## Abstract

This review addresses the current status of drug therapy for the management of fibromyalgia syndrome (FMS) and is based on interdisciplinary FMS management guidelines, meta-analyses of drug trial data, and observational studies. In the absence of a single gold-standard medication, patients are treated with a variety of drugs from different categories, often with limited evidence. Drug therapy is not mandatory for the management of FMS. Pregabalin, duloxetine, milnacipran, and amitriptyline are the current first-line prescribed agents but have had a mostly modest effect. With only a minority of patients expected to experience substantial benefit, most will discontinue therapy because of either a lack of efficacy or tolerability problems. Many drug treatments have undergone limited study and have had negative results. It is unlikely that these failed pilot trials will undergo future study. However, medications, though imperfect, will continue to be a component of treatment strategy for these patients. Both the potential for medication therapy to relieve symptoms and the potential to cause harm should be carefully considered in their administration.

## 

*The desire to take medicine is perhaps the greatest feature which distinguishes man from animals*.

Sir William Osler (1849–1919)

## Introduction

Roughly 2% of the developed world’s population meet either the 1990 or 2010 American College of Rheumatology criteria for fibromyalgia syndrome (FMS) [1-5]. Patients with FMS report a wide array of somatic and psychological symptoms, and each contributes to a varying degree of symptom burden and functional disability [6,7].

Many factors shape the modern practice of FMS pharmacologic therapy. Understandably, patients seek symptom relief, and prospective studies [8] and consumer reports demonstrate considerable use of pharmacological and non-pharmacological therapies [9,10]. Some may even hope for complete eradication of symptoms, a goal that currently is mostly unattainable. Physicians are trained to alleviate symptoms, using available evidence and clinical experience, even in the absence of a cure. Drug prescription has been the foundation of medical care over the past several decades, driven by the success of many pharmacologic interventions for various medical conditions. This success has positioned drug prescription at the center of the practice of medicine and has become entrenched in patient expectations for medical care [11]. Pharmaceutical companies are also highly motivated to provide FMS patients with successful, but profitable, pharmacological therapies. Identifying an effective FMS drug would be a triumph for patients, health-care providers, and industry alike, and a potential financial market is valued at $1.2 billion in the US alone [12]. Therefore, the status of FMS drug therapy reflects the needs of these various stakeholders, and each has a distinct agenda. In this review, we address the following questions:

• Which drugs are considered standard for the treatment of FMS?

• What are the potential benefits and harms of standard treatment drugs in FMS?

• Are there any other commonly prescribed FMS treatments whose use is supported by (limited) evidence?

• Are there any other commonly prescribed FMS treatments whose use is not supported by evidence?

• Are there any drugs not recommended for use in the treatment of FMS?

• Which drugs held promise but without success? Are there new hopes for a ‘magic bullet’ for FMS?

• Are any particular drugs better than the others?

• What should physicians and patients be mindful of when drug therapy is considered?

## Review

### Methods

Our analyses and recommendations are based on the following sources:

#### ***a. Interdisciplinary guidelines***

Interdisciplinary FMS management guidelines have recently been developed in Canada [13] and Germany [14]. The German guideline was based on a systematic search of the literature from inception to December 2010. The strength of recommendations was developed by multiple-step formal procedures to reach a consensus. As measure of efficacy, standardized mean difference of drugs versus control group in randomized controlled trials (RCTs) was chosen for the outcomes of pain, fatigue, sleep problems, and health-related quality of life (HRQOL) at final treatment and, if available, at follow-up. Dropout rates for the active versus control group were chosen as a measure of tolerability. Adverse events as reported in RCTs, open-label studies, and case reports were chosen as measure of risks. Efficacy, tolerability, and risks and applicability of therapies available were summarized in a balance sheet [15,16]. Similarly, the Canadian guideline was based on a systematic literature search directed by questions derived from a needs assessment. The literature was evaluated for level of evidence according to a standard method; recommendations were formulated and reviewed by a multidisciplinary group and, after a voting procedure, assigned a level of recommendation [13].

#### ***b. Meta-analyses***

Cochrane reviews on antidepressants [17-20] and anticonvulsants [21,22] in FMS were accessed. If the number of responders (for example, 30% pain reduction) was not reported, a validated imputation method to calculate pain reduction rates from reported means and standard deviations was used [20,23].

#### ***c. Observational studies***

The external validity of drug therapy in FMS is severely limited because of uniform exclusion of inflammatory rheumatic diseases and DSM-IV (*Diagnostic and Statistical Manual of Mental Disorders, Fourth Edition*)-defined psychological disorders from studies [16]. Therefore, we refer to the results of FMS consumer reports [9,10], of cohort studies of patient databanks [24], and of administrative claims data [25] to better understand patterns of drug use in the general population.

### Drugs currently prescribed as standard treatment for fibromyalgia syndrome

The tricyclic antidepressants (TCAs), particularly amitriptyline, were the mainstay of FMS pharmacotherapy up to the last decade. Although amitriptyline has never received approval for treatment of FMS, it is available in most countries worldwide, relatively cheaply, and is approved for treatment of depression or chronic neuropathic pain syndromes. Originally amitriptyline was thought to act by reducing alpha intrusion into non-rapid eye movement (non-REM) sleep, but the current understanding suggests an effect on pain modulation via serotonin and norepinephrine. Subsequent study identified the efficacy of pregabalin (an anticonvulsant with α2-δ ligand binding) and two newer antidepressants - the serotonin norepinephrine reuptake inhibitors (duloxetine and milnacipran) - in relief of FMS symptoms. All three latter agents are approved for FMS therapy by the US Food and Drug Administration (FDA). Pregabalin is approved for FMS therapy not only in the US and Canada but in a number of countries in South America, the Middle East, and Asia.

Duloxetine is approved in 25 countries besides the US, whereas milnacipran is approved in the US, Argentina, Australia, and South Korea. In contrast, the European Medical Agency (EMA) denied approval of these three drugs on the grounds that they had not shown superiority to placebo in pain reduction in studies that included European patients [17,22]. All three are approved for the treatment of anxiety or depressive disorders or both in some European countries.

#### ***Efficacy and tolerability***

The pain-reducing benefits of pregabalin, duloxetine, and milnacipran are minimally outweighed by side effects (Table 1). In that only a minority of patients will experience substantial relief [17,19,20], vigilance regarding adverse effects is required (Table 2). Most commonly, tolerability is limited by troublesome, but generally not serious, side effects, such as drowsiness, weight gain or peripheral edema for pregabalin, and gastrointestinal or cognitive intolerance for duloxetine and milnacipran.

**Table 1:** Thirty percent pain reduction rates in randomized controlled trials with antidepressants and pregabalin in patients with fibromyalgia syndrome

**Drug (reference)**	**Number of RCTs/participants**	**30% pain reduction true drug vs. placebo (%)**	**RR 30% pain reduction (95% CI)**	**Dropout rate due to adverse events, percentage**	**RR dropout rate due to adverse events (95% CI)**
Duloxetine^a^[17]	5/1,884	46.8 vs. 34.0	1.33 (1.18-1.51)	18.7 vs.10.4	1.65 (1.30-2.09)
Milnacipran^b^[17]	5/4,110	36.4 vs. 28.1	1.38 (1.25-1.51)	21.5 vs.11.0	2.00 (1.47-2.73)
SSRIs^c^[20]	7/414	36.4 vs. 20.6	1.59 (1.01-2.52)	9.5 vs. 7.0	1.60 (0.84-3.04)
TCAs^d^[20]	9/542	48.3 vs. 27.8	1.60 (1.15-2.34)	5.2 vs. 6.5	0.84 (0.46-1.52)
Pregabalin^e^[22]	5/3,259	40.0 vs. 29.1	1.37 (1.22-1.53)	19.4 vs. 11.0	1.68 (1.36-2.07)

^a^Dosages 60 mg/day, 120 mg/day, and 60 to 120 mg/day flexible pooled together. ^b^Dosages 100 mg/day, 200 mg/day, and 100 to 200 mg/day flexible pooled together. ^c^Citalopram 20–40 mg/day, Fluoxetine 12–80 mg/day, Paroxetine 20–60 mg/day. ^d^Amitriptyline 10–50 mg/day. ^e^Dosages 150 mg/day, 300 mg/day, 450 mg/day, 600 mg/d, and 300–450 mg/day flexible pooled together. One study with 556 participants and an enriched enrolment withdrawal design could not be included in meta-analysis. CI, confidence interval; RCT, randomized controlled trial; RR, relative risk; SSRI, selective serotonin reuptake inhibitor; TCA, tricyclic antidepressant.

**Table 2:** Summary of the US Food and Drug Administration’s contraindications and warnings of pregabalin and antidepressants

**Drug**	**Contraindications**	**Warnings and precautions**	**Last update**
Pregabalin	1. Known hypersensitivity to pregabalin or any of its components	1. Suicidal behavior and ideation	June 2011
		2. Usage in pregnancy	
		3. Hypersensitivity reactions	
		4. Angioedema	
		5. Peripheral edema	
		6. Dizziness and sleepiness with impairment of the ability to drive or operate machinery	
		7. Gynecomastia and breast enlargement	
Duloxetine	1. Concomitant use of monoaminooxidase inhibitors	1. Suicidality	Jan. 2010
	2. Uncontrolled narrow-angle glaucoma	2. Hepatotoxicity	
	3. Substantial alcohol use or evidence of chronic liver damage	3. Orthostatic hypotension and syncope	
	4. Severe renal impairment	4. Serotonin- or neuroleptic syndrome-like reactions	
		5. Abnormal bleeding	
		6. Discontinuation syndrome	
		7. Activation of mania	
		8. Blood pressure control	
		9. Hyponatremia	
		10. Glucose control in diabetes	
		11. Slow gastric emptying	
		12. Urinary hesitation and retention	
Milnacipran	1. Concomitant use of monoaminooxidase inhibitors	1. Suicidality	Jan. 2009
	2. Uncontrolled narrow-angle glaucoma	2. Hepatotoxicity	
	3. Substantial alcohol use or evidence of chronic liver damage	3. Serotonin syndrome	
		5. Abnormal bleeding	
		6. Discontinuation syndrome	
		7. Elevated blood pressure	
		8. Urinary hesitation and retention	
		9. Seizures	
Tricyclic agents	1. Prior hypersensitization	1. Suicidality	Amitriptyline Jan. 2010
	2. Concomitant use of monoaminooxidase inhibitors	2. Anxiety and insomnia	
	3. Acute recovery phase following myocardial infarction	3. Activation of mania/hypomania and schizophrenia	
		4. Cardiovascular disorders	
		5. Hyperthyroid patients or those receiving thyroid medication	
		6. Elective surgery	
		7. Elevated or lowered blood sugar	
		8. Impaired liver function	
Serotonin reuptake inhibitors	1. Concomitant use of monoaminooxidase inhibitors, thioridazine and pimozide	1. Suicidality	Fluoxetine April 2011
		2. Abnormal bleeding	
		3. Anxiety and insomnia	
		4. Activation of mania/hypomania	Paroxetine July 2011
		5. Hyponatremia	
		6. Seizures	

Although life-threatening side effects such as serotonin syndrome and liver failure with antidepressants [17] and heart failure with pregabalin [22] are very rare, they should be kept in mind. Pregabalin abuse has also been reported in susceptible populations, leading to classification as a Class V controlled substance in the US [26].

The benefit-risk balance seems to be modestly favorable for TCAs and essentially equal for selective serotonin reuptake inhibitors (SSRIs) (Table 1), but the quality of evidence for TCAs and SSRIs is relatively poor [19,20]. A direct comparison of TCA and SSRIs to pregabalin and serotonin noradrenaline reuptake inhibitors (SNRIs) is flawed for a number of reasons. The data on TCAs and SSRIs are based on early studies conducted between 1986 and 1998, generally with small sample sizes, whereas data for pregabalin and SNRIs are more robust, performed with much larger sample sizes in studies designed to seek therapeutic approval conducted between 2004 and 2010. Study design has improved considerably over the years as application of rigorous scientific methodology has increased [20]. Primary endpoints have also changed with more recent attention to global health status rather than focusing on any single particular symptom. And finally, both the placebo as well as the nocebo (dropout) response rate have increased over time [27,28]. Therefore, direct comparison of TCAs and SSRIs with pregabalin or SNRIs is confounded.

#### ***Effectiveness***

Although RCTs can provide discrete estimates of effect, the effectiveness of a drug is likely best characterized by the real-life experience provided by observational studies in the general population, independent of industry and reliant on clinical judgment. TCA use in patients with newly diagnosed FMS was examined by using privately insured US administrative claims data, covering 1999 to 2005. The mean (median) duration of the first treatment episode was 150 (58) days, with 60.8% augmenting TCA use with other drugs, 61.8% switching to another drug at the end of their TCA episode, and 22.8% discontinuing TCAs without switching [25]. Therefore, TCAs represent limited efficacy as a single drug in the clinical setting. Similarly, treatment information was provided in an 11-year follow-up of about 3,123 US adult FMS patients registered in the National Data Bank of rheumatic disease. The centrally acting agents (pregabalin, gabapentin, duloxetine, and milnacipran) received approval during the observation period of the study. Use rates increased from 10% to 39% over the 11 years, but mean pain, fatigue, and disability measurements did not change in this study population. For patients treated with duloxetine or milnacipran, or pregabalin, pain scores were reduced significantly - by 0.17 (0.03, 0.30) units, an improvement of 2.8% - following the start of these drugs but with no significant improvements in fatigue or function. These results question whether the changes attributable to use of these agents are truly clinically meaningful. However, a patient’s choice to continue a treatment implies some level of satisfaction. The estimated 25th and 50th percentiles of time to discontinuation for centrally acting agents were 1 and 2.5 years, respectively [24]. In a German fibromyalgia consumer reports study, patients did not identify any medication in the top 10 effective therapies. Rather, medication therapy was only perceived as harmful, with pregabalin identified as the 3rd, duloxetine the 6th, and amitriptyline the 7th most harmful therapies [10]. Taking all these factors and, especially, real-world clinical observation into consideration, we contend that the overall benefit of these agents remains limited for most patients.

### Other commonly prescribed fibromyalgia syndrome treatments with use supported by (more limited) evidence

#### ***Tramadol***

Tramadol, a weak μ-opioid receptor agonist and a reuptake inhibitor of serotonin and norepinephrine, is frequently used in FMS treatment [9,10]. As this is the only opioid medication that has been studied in FMS, it is unfortunate that the quantity of evidence is small. Studied in 313 patients with FMS, tramadol/acetaminophen was superior to placebo over 12 weeks in pain reduction and improved quality of life [29]. Sixty-nine percent of 100 patients tolerated tramadol and achieved benefit during the open phase. The responders were randomly assigned: after 3 weeks, tramadol was superior to placebo in the reduction of pain but not in measures of HRQOL [30]. Therefore, tramadol may be considered a step up from over-the-counter simple analgesics for pain relief but has a side effect profile similar to opioid agents but generally less severe. No studies to date have examined the efficacy of tapentadol, a drug with effects somewhat similar to those of tramadol, but with mostly norepinephrine and opioid agonist effect.

#### ***Cyclobenzaprine***

Cyclobenzaprine, a muscle relaxant that is structurally similar to the TCAs, is frequently used in North America [9] but is unavailable in most European countries. A meta-analysis of five RCTs with a total of 392 patients conducted in the 1990s demonstrated that cyclobenzaprine-treated patients were three times more likely to report overall improvement and moderate reductions in individual symptoms of pain and sleep problems compared with placebo after 4 to 24 weeks [31]. A recent RCT with 36 patients demonstrated superiority of low-dose cyclobenzaprine over placebo for improving sleep after 8 weeks [32]. Therefore, cyclobenzaprine may be considered a treatment option, but unfortunately evidence is limited.

### Other prescribed fibromyalgia syndrome treatments with use not supported by evidence

A wide array of drugs has failed to show superiority over placebo (Table 3). It should be noted that these drugs were examined mostly in either a single or few studies and with small sample sizes.

**Table 3:** Drug classes that failed to show superiority over placebo in reducing fibromyalgia syndrome symptoms

**class (drug)**	**Number of studies /participants/duration of trial in weeks**
Antiviral agents (valacyclovir)	1/30/6
Anxiolytics	
Alprazolam	1/31/8
Bromazepam	1/84/8
Dopamine agonists	
• Pramiprexole	1/60/12^a^
• Ropirinol	1/181/12
• Terguride	1/99/12
Hormones	
Calcitonin	1/11/4
Dehydroepiandrosteron	1/52/4
Prednisone	1/20/2
Hypnotics	
Zolpidem	1/14/8
Interferon	1/28/4
Ketamine (intravenous)	1/20/0,5
Local anesthetics (intravenous): Lidocaine	3/177/4
Neuroleptics: Ritanserin	1/24/16
Serotonin receptor antagonists	
Tropisetron	2/261/1
Odansetron	1/42/1

A pooled analysis was conducted in case of more than one study [16]. ^a^Pramiprexole was superior to placebo [33].

### Prescribed drugs that are not recommended for use in the treatment of fibromyalgia syndrome?

#### ***Strong opioids***

Worldwide, patients with FMS are prescribed strong opioids in the absence of any published RCTs examining the efficacy in FMS. With increasing concerns of the personal and societal risks of opioid treatment for non-cancer chronic pain conditions, this trend in opioid use is concerning. In a nationally representative US dataset of commercially insured individuals (245,758 patients with FMS), 11.3% (4% to 20%) received chronic daily opioid therapy [34]. Similarly, in a study of about 7 million members of a German statutory health insurance company, strong opioids were prescribed for 11% with FMS [35]. The German guideline search of literature identified only one case series wherein the majority of patients worsened or discontinued therapy because of side effects [36]. Opioid use in FMS was associated with negative health-related measures in a prospective cohort study of a Canadian interdisciplinary pain center [37]. Strong opioids were ranked as the number one most harmful therapy in the German FMS consumer reports [10]. The Canadian [13] and German [15] guidelines unanimously strongly discouraged the prescription of strong opioids on the basis of lack of evidence in the context of patient-related side effects and risks to society with opioid prescription abuse. US authors [38] rationalized that chronic opioid use is inappropriate in the treatment of FMS because of the interaction of unique pathophysiologic characteristics of FMS patients and effects associated with chronic opioid use. The common practice of prescribing short-acting narcotics on an ‘on demand’ basis to handle sudden increases in painful symptoms has not yet been addressed in either studies or practice guidelines but is generally not recommended in guidelines for the treatment of chronic, non-cancer pain [39].

#### ***Nonsteroidal anti-inflammatory drug***

Nonsteroidal anti-inflammatory drug (NSAID) use is prevalent for patients with FMS, either as a prescription medication or as an on-demand over-the-counter preparation. Taking into consideration the increasing knowledge of risks associated with chronic NSAID use, caution should be exercised when advising patients with FMS. Forty-one percent of the participants of the German FMS consumer reports were current users of NSAIDs [10], whereas in the US, 36% report current use of ibuprofen [9]. The German [15] guideline gave negative treatment recommendations for NSAIDs on the basis of lack of superiority compared with placebo after 1 to 8 weeks in four RCTs with a total of 181 patients and also because of potential side effects (for example, gastrointestinal bleeding and cardiovascular risks) for long-term use. However, in the US [9] and German [10] consumer reports, a moderate benefit was attributed to NSAIDS, mainly by patients with self-reported osteoarthritis and inflammatory rheumatic diseases [10]. It is noteworthy that the latter patients were excluded in nearly all drug studies in FMS, but fibromyalgia symptoms are prevalent in these conditions. The primary use for NSAIDs remains for the management of pain in rheumatic disease [40].

### Drugs that held promise but without success

Sodium oxybate, an agent that affects dopamine release by binding to the GABA_B_ and gamma-hydroxybutyric acid receptors, demonstrated efficacy in RCTs for FMS symptoms [28]. However, this agent was denied approval by the FDA on the grounds of safety and concerns about diversion. Sodium oxybate was similarly refused approval by the EMA as short- and long-term efficacy had not been demonstrated in the EU population. The safety profile of sodium oxybate is unfavorable with a high frequency of central nervous system-related adverse events, psychomotor effects that can pose a risk of motor vehicle accidents, abuse, and diversion, and the societal risk related to use as a date-rape drug [28].

Serotonin receptor agonists, such as tropisetron, have been studied in four European RCTs, but a meta-analysis demonstrated no significant superiority over placebo [16]. The pharmaceutical companies of these drugs have halted further trials (Späth, 2012, personal communication).

### Hope for the magic bullet?

#### ***Cannabinoids***

Cannabinoid molecules have analgesic as well as sleep-promoting effects. Nabilone, a synthetic tetrahydocannabinol, has been tested in two small studies. In a 2-week crossover study of 32 patients, nabilone was superior to amitriptyline for reduction of sleep problems but without differences in pain or quality of life [41]. In the second study, nabilone was superior to placebo after 4 weeks in 40 patients for both pain and quality of life [42]. There were frequent side effects, including vertigo (47%), dizziness (35%), and nausea (31%). Nabilone did not receive recommendation by the German guideline [15], because of abuse potential [43]. The Canadian guideline gave a weak recommendation for a trial of pharmacologic cannabinoid, particularly in case of sleep disturbance [13].

#### ***Growth hormone***

Three RCTs of growth hormone (two studies compared with placebo, one as add-on to multicomponent therapy including antidepressants and tramadol) of 157 total patients demonstrated benefits of growth hormone for pain and fatigue after 9 to 18 months [44-46]. Cost and potential side effects (metabolic changes, carpal tunnel syndrome, and anemia) raise concerns about its use.

#### ***Quetiapine***

Four double-blind controlled studies have explored the efficacy of quetiapine, either alone or as an add-on treatment, and only one study has been published to date. The current available evidence suggests that quetiapine may be useful, prompting further study [47]. In view of side effects associated with the atypical neuroleptics such as weight gain and metabolic changes [48], serious concerns regarding their long-term use remain.

#### ***Naltrexone***

In a randomized, double-blind, placebo-controlled, counterbalanced, crossover study with 31 patients with FMS, low-dose naltrexone was superior to placebo in the reduction of pain and depressed mood but not in the reduction of fatigue and sleep problems [49]. Parallel-group RCTs with larger sample sizes are needed to fully determine the efficacy of this medication.

#### ***And many others***

A search in ClinicalTrials.gov on 25 May 2013 revealed studies that are active or completed with drug classes such as antidepressants (agomelatine, paroxetine, and trazodone), cannabinoids, dopamine agonists (droxidopa), hormones (low-dose hydrocortisone), and hypnotics (eszopilcone) as well as with new drug classes such as AD337 (centrally acting non-opioid analgesic) and neurotropines. Overall, it appears that many drugs are currently undergoing testing of their efficacy in FMS. To date, all agents under testing with available data show limited promise at this time, and efficacy appears to be similar to that seen with currently available agents.

### Are any particular treatments better than the others?

The Oregon Health & Science University performed a systematic review of comparative therapy through October 2010 by using published data, FDA medical and statistical reviews, and dossiers submitted by pharmaceutical companies. The authors found 47 eligible studies. Head-to-head trials were few, and the evidence provided was weak. Short-term treatment with immediate-release paroxetine was superior to amitriptyline in reducing pain and sleep disturbance, and amitriptyline was equivalent to cyclobenzaprine and nortriptyline. Withdrawals due to adverse events were similar. Using indirect-comparison meta-analysis, the authors reported weak evidence that there were differences between drugs for particular symptoms. Duloxetine was superior to milnacipran on outcomes of pain, sleep disturbance, depressed mood, and HRQOL. Both duloxetine and milnacipran were superior to pregabalin for improvement in depressed mood, whereas pregabalin was superior to milnacipran for improvement in sleep disturbance. Amitriptyline was similar to duloxetine, milnacipran, and pregabalin on outcomes of pain and fatigue, and data on the other outcomes were insufficient. Although there were differences in specific adverse events, they did not produce any differences in overall withdrawals, adverse events, or withdrawals due to adverse events [50].

Nüesch and colleagues [51] performed a systematic search of the literature up to December 2011, including 102 trials with 14,982 subjects and eight active interventions (TCAs, SSRIs, SNRIs, pregabalin, aerobic exercise, balneotherapy, cognitive behavioral therapy, and multicomponent therapy) and examined the data by a network analysis. Methodological quality and small numbers introduced heterogeneity and inconsistency in this analysis. When the analysis was restricted to large trials with at least 100 patients per group, heterogeneity was low and benefits for SNRIs and pregabalin compared with placebo showed statistical significance but only limited clinical relevance [51].

### What should physicians and patients be mindful of when drug therapy for fibromyalgia syndrome is considered?

Drug therapy is not a panacea for the care of patients with symptoms of FMS. For many, who may have been through various treatment trials, the final compromise may be the limited use of prescription medications, on demand over-the-counter agents, and focus toward non-pharmacologic strategies. In those continuing drug treatments, many will use a combination of drugs, generally in lower doses than may be recommended by manufacturers. There is, however, no current evidence that patients benefit from drug combinations, despite widespread use.

The best-studied drugs for treatment of FMS are amitriptyline, pregabalin, duloxetine, and milnacipran, leading to a recommendation as first-line treatment options by two recent evidence- and interdisciplinary consensus-based guidelines on FMS [13,14]. In general, the data on their efficacy are robust even though the average incremental benefit over placebo is small [16,17,19-22]. The evidence for amitriptyline’s beneficial effect is not as substantial as the others but should be taken in context with decades of its perceived success in FMS treatment [19]. At this time, the data suggest that there are not substantial differences in efficacy between duloxetine, milnacipran, and pregabalin. Rather, the evidence suggests that the majority of medications can provide an improvement in pain of 30% in half of the patients taking the medication and that improvements in pain of 50% are seen in a third of patients. These observed benefits do not translate into global improvement in well-being as measured by Short Form Health Survey-36 (SF-36) or the Health Assessment questionnaire. Despite treatment, physical health perceptions remain substantially lower (1.5 standard deviations) in FMS than in the general population, and scores of patients with FMS are essentially equivalent to those seen in patients on chronic dialysis [52]. Cyclobenzaprine, other SSRI antidepressants (fluoxetine and paroxetine), and tramadol with and without acetaminophen can be regarded as second-line treatment options. Therapies with other drugs (for example, nabilone and quetiapine) remain experimental at this time.

Drug therapy should be initiated with small dosages and with gradual upward titration. We recommend starting amitriptyline 10 mg at night, pregabalin 50 to 100 mg at night, duloxetine 30 mg daily, and milnacipran 50 mg in the morning. The highest recommended dosages are amitriptyline 10 to 50 mg/daily, pregabalin 300 to 450 mg/daily, duloxetine 60 mg/daily, and milnacipran 100 mg/daily. Patients should be monitored regularly for efficacy and tolerability, whether by visit, by phone, or by email, especially within the first weeks of treatment. Responses should be expected within 2 to 4 weeks once recommended dosage has been achieved [21]. Treatment should be continued in treatment responders only. Medications that are not providing the patient benefit should be discontinued rather than supplemented by other drugs in pursuit of higher benefits by ‘combining’ drug treatments.

Contrary to popular perception, drug treatments should be recommended with reservation because of limited efficacy and side effect potential. Though not supported by RCTs, two recent guidelines strongly discourage drug therapy as a single management strategy for FMS. Drug therapy may be added to self-management strategies that include aerobic exercise or psychological therapies (or both) but with the ideal objective that long-term management will be achieved with no or minimal drug therapy [13,14]. Shared decision-making by patients and physicians is required to provide optimal health care for patients with FMS. Drug choices should target the most prominent comorbid symptoms. Amitriptyline or pregabalin could be preferred for those with sleep disturbances, duloxetine for major depression, and duloxetine or pregabalin for general anxiety disorder [17,20,22]. Patients with comorbid rheumatic disease may consider tramadol or either duloxetine or tramadol for comorbid osteoarthritis. Potential side effects (for example, sexual dysfunction by SSRI and weight gain by TCAs and pregabalin) and contraindications (for example, SNRIs in case of severe liver damage and pregabalin for professional drivers) should be reviewed and carefully considered. Medication cost and local status of approval may be an important issue, in particular in choosing between generic amitriptyline and the other patented first-line agents [20].

Both physicians and patients should have realistic expectations about the potential benefit of these drugs. Although patients may initially experience symptom relief with good tolerance, the majority will ultimately discontinue therapy because of inadequate response or unacceptable side effects [17,21,22]. Reduced dosing for pregabalin may allow better tolerability, although this generally does not apply for duloxetine or milnacipran. Taking alpha-2-delta ligands at night or SNRIs with food and at a low initial dose helps improve tolerability. Placebo and nocebo responses play an important role in the positive and negative (dropout rate) effects of drugs in FMS, and estimates are that these effects account for up to 60% of measured drug efficacy and harms [27,28]. The deliberate use of psychological strategies underlying the placebo response, such as promoting positive treatment expectations and establishing a positive therapeutic relationship and regular health-care contact, can likely bolster the positive effects of drug treatment. Similarly, open discussion of previous drug experiences, exploring potential unrealistic fears, and regular patient contact may attenuate the nocebo response. Important points to consider in the drug therapy of FMS-patients are summarized as follows:

• Drug therapy is not mandatory.

• Shared decision-making for or against drug therapy

• Tailored selection of drugs according to

– Key symptoms beyond pain (fatigue, sleep problems)

– Psychological comorbidities (depressive or anxiety disorder or both)

– Physical comorbidities (rheumatic disease)

– Contraindications

– Individual importance of frequent side effects (for example, weight gain)

• Augment placebo and reduce nocebo response.

• Start low, go slow.

• Monitor for efficacy, tolerability, and safety.

• Progressive treatment reduction in responders

• Consider drug holidays.

• Promote long-term drug-free self-management of the patient.

## Conclusions

Drug therapy as the sole strategy for the management of patients with FMS should be discouraged. Taking into consideration the modest effect of currently available drugs, high prevalence of adverse effects, and poor record of continued use, the health-care community must be vigilant in adhering to responsible prescribing practices and carefully monitor patients for both efficacy and side effects.

### Note

This article is part of the series on New perspectives in fibromyalgia, edited by Daniel Clauw. Other articles in this series can be found at http://arthritis-research.com/series/fibromyalgia

## Abbreviations

EMA: European Medical Agency; FDA: US Food and Drug Administration; FMS: Fibromyalgia syndrome; HRQOL: Health-related quality of life; NSAID: Nonsteroidal anti-inflammatory drug; RCT: Randomized controlled trial; SNRI: Serotonin noradrenaline reuptake inhibitor; SSRI: Selective serotonin reuptake inhibitor; TCA: Tricyclic antidepressant.

## Competing interests

WH has received one consulting honorarium from Daiichi-Sankyo (Tokyo, Japan) and honoraria for educational lectures from Abbott Germany (Wiesbaden, Germany), Eli Lilly and Company (Indianapolis, IN, USA), Janssen-Cilag (Neuss, Germany), and Pfizer Inc. (New York, NY, USA) in the last 5 years. He is the head of the steering committee of the German guideline on FMS and a member of national and international scientific societies of internal medicine, pain medicine, psychosomatic medicine, and sports medicine. He is an active participant in Cochrane Collaboration reviews on efficacy of drugs and cognitive behavioral therapies in FMS. M-AF has received consulting fees, speaking fees, and/or honoraria from Biovail (Mississauga, ON, Canada), Forest Laboratories (New York, NY, USA), Janssen-Cilag, Eli Lilly and Company, Pfizer Inc., Purdue University (West Lafayette, IN, USA), and Valeant (Laval, QC, Canada). She is the head of the steering committee of the Canadian guideline on FMS. BW has received one consulting honorarium from Jazz Pharmaceuticals (Dublin, Ireland) in the last 5 years. He is an active participant in Cochrane Collaboration reviews of FMS drug efficacy and a member of the American College of Rheumatology. CS has received consulting honoraria from Pfizer Inc. and Eli Lilly and Company and speaker honoraria from Allergan (Irvine, CA, USA), Baxter Inc. (Deerfield, IL, USA), CSL Behring (King of Prussia, PA, USA), Eli Lilly and Company, Genzyme Corp (Cambridge, MA, USA), GlaxoSmithKline (Uxbridge, Middlesex, UK), and Pfizer Inc. She is an active participant in Cochrane Collaboration reviews on efficacy of drugs in FMS.
